# Aims and Uses of a Medical Society1Annual address of the president of the Buffalo Academy of Medicine, June 13, 1905.

**Published:** 1905-10

**Authors:** Arthur W. Hurd

**Affiliations:** Buffalo, N.Y., Superintendent of Buffalo State Hospital


### BUFFALO MEDICAL JOURNAL.

VOL. LXI. OCTOBER, 1905. NO. 3

### ORIGINAL COMMUNICATIONS.

### Aims and Uses of a Medical Society.[1]

BY ARTHUR W. HURD, A. M., M. D., Buffalo, N. Y.,
Superintendent of Buffalo State Hospital.

WITH a view to presenting certain considerations which may prove helpful in extending the scope and usefulness of the Academy of Medicine, I have been led to study some of the aims and uses of a medical society. Such an organization, as I take it, should bear certain relations to the profession first, and to the community second.

A brief review of what our academy has done during the past year will enable us to get a better perspective. I find that we have held during the past year thirty-two meetings. Of these, there have been thirty held by the surgical, medical, pathological and obstetrical sections, and two were special meetings. Of the thirty meetings of the regular sections, three were "stated" ones. These sessions have direct reference to the medical profession and the study of medical science. The two special meetings have a bearing upon the relations of the society to the community. Of the papers which have been read and discussed in the regular meetings, the topics have embraced a wide range. The essayists have numbered fifty-six; of this number eight have been men of distinction from outside of Buffalo. Of the subjects presented, internal medicine embraced about 46 per cent., surgery about 24 per cent., pathology about 15 per cent., obstetrics and gynecology about 15 per cent., making a total of 100 per cent.

As to the relations and uses of the society in regard to the profession, since I have had this subject under consideration, there has been sent to me a paper on the educational value of

1. Annual address of the president of the Buffalo Academy of Medicine, June 13, 1905.

the medical society, by Dr. William Osler, which was read before the New Haven County Society. In it there is reference to the organization of the Litchfield County (Connecticut) Medical Society, which has for me a particular ancestral interest. I think that the statement of the conception of the uses of a medical society, entertained by our good medical forefathers in 1778 is not out of place. This statement follows:

This society was formed on the most liberal and generous principles, and was designed first to lay a foundation for that unanimity and friendship which is essential to the dignity and usefulness of the profession; to accomplish which, they resolved, secondly, to meet once in three months; thirdly, that in all cases where counsel is requisite they will assist each other without reserve; fourthly, that all reputable practitioners in the country, who have been in the practice for one year or more, may be admitted members; fifthly, that they will communicate their observations on the air, seasons and climate, with such discoveries as they may make in physic, surgery, botany or chemistry, and deliver faithful histories of the various diseases incident to the inhabitants of this country, with the mode of treatment and event in singular cases; sixthly, to open a correspondence with the medical societies in the neighboring states and in Europe, for which purpose they have a standing committee of correspondence; seventhly, to appoint a committee for the purpose of examining candidates for the profession, and to give certificates to the deserving.

The progress of events has rendered unnecessary the examination of candidates for entrance into the profession, and "correspondence with medical societies of neighboring states and Europe," but aside from these, their conception of the purposes of a medical society seems to me as applicable now as then. Our medical colleges provide for the education of the student until he reaches the point of graduation. A foundation is given the graduate,—a diploma entitling him to practise. This is in many cases supplemented by hospital appointment and training, and there is no physician, I take it, even though but recently graduated, and certainly not one among those who have supplemented their college course by a hospital training, who will for a moment contend that their medical education has ended with graduation. In fact, we regard it as being but fairly begun, and to the studious, that which takes the place of an educational force after graduation, aside from books and journals, must be the post-graduate course; either a school or a society. But books and journals while having their place, however, do not give that subtle something which comes with the friction of mind with mind, and the interchange of practical experiences. To my mind a most potent means for the physician once out of college to "keep up," to be refreshed and to acquire progress in medicine, is by membership and attendance and participation, in a good, live medical society. I say this believing that the sober judgment of the profession will bear me out. No two cases of sickness are alike; cases from the authorities may never just correspond with the patients you have seen this afternoon, but the experience of brother practitioners in the same locality who are working on the same class of cases, is useful and enlightening. The exhibition of cases forms in itself a clinic which may be of inestimable value. The paper from which I quoted states that at the International Congress in London in 1881, there was presented at the Clinic Museum a group of cases of myxedema. There were many men present from all parts of the world, and the general recognition of the disease outside of England dates from that meeting. It is one of these cases where to see is almost to learn the diagnosis, and a description no matter how good, fails to give a true picture. How many similar cases of rarer conditions can be learned by just such a clinical demonstration in a medical society; for we must remember that for few, especially outside the cities, is there time and opportunity afforded to attend the clinics in the medical colleges. I do not refer here, or speak in commendation of any long-winded, tedious presentation of histories of cases with unimportant details, but of essentially important points of cases, even of common occurrence, and the consideration of scientific, modern, up-to-date methods of diagnosis cannot help but be of the greatest advantage. There are 687 physicians in Buffalo. Of this number 197 appear to appreciate the advantages of an academy of medicine, but if even a majority of this membership should make it a point to attend with a certain degree of regularity, our meetings would gain much in interest and value.

When a young man begins the practice of medicine there is a "stand and wait" period or better, a "sit and wait" period, which should be most zealously employed in preparation both scientifically and socially for the time of greater professional activity which should normally follow. From this patientless beginning, up to and including the so-called "forty-visit" a day man, there is constant need of study and work if one is to keep up with the tremendous strides in medicine. I am not sure but the busy and successful practitioner needs the help of the medical society more than the fledgling. The very busy man needs the daily help of the laboratory and advanced scientific methods of diagnosis, if he is to do good work. If he has not the time or training to do it himself he should take the time and acquire the training, or at least acquire such a discriminating knowledge as will lead him to know when and what he requires in laboratory work on the part of others. The art of medicine is important, but in these days of scientific accuracy in diagnosis, the microscope and test tube and *x*-ray, cannot be ignored even by the busiest of men, as by their careful use mistakes are prevented and injustice to our clientage avoided. It is in journals and books and medical societies that knowledge and familiarity with what is now in medicine must be learned, and wise is the doctor who early discerns that a membership in an active medical association is one of the best means of keeping in touch with the best, both of science and of men that is worth knowing in his profession.

"A foundation for the unanimity and friendship which is essential to the dignity and usefulness of the profession." What better statement could we make now more than a hundred years since, than was written of the dignity which our profession would hold before the public, and its usefulness as well, did the profession possess the unanimity and friendship here spoken of. It seems but necessary to mention it to recognise it. How often do we find that the man whom we thought from the rumors of disgruntled patients, or envy of rival practitioners to be a man of "hoofs and horns," in reality, when we come to meet him personally is a gentleman, both professionally and socially. The medical society becomes a clearing house where we not only acquire new medical knowledge, but also a better and higher opinion of our fellow practitioners. How easy it is to believe evil of those whom we know only by hearsay or reputation, and how highly do we think of those whom we know the best and how slow are we to believe slander and gossip about those with whom we are well acquainted. Acquaintance engenders knowledge and sympathy. Sympathy reveals community of interest,—community of interest fosters organisation, and organisation brings power and influence, and this brings me to the consideration of another of the uses of a medical society,—namely, its relation to and influence on the community. We have a duty to our patients, but we have a duty to the community in which we live, not only as physicians but as citizens. In sanitary matters, in milk supply, in food inspection, in sewerage and water supply, in education, in our public schools, in municipal improvement and adornment, in the immigration problem, in the care of the dependent and defective classes, the medical society should, and in many instances does, take the leading part in guiding and influencing public sentiment. As instances of this, I may mention the work of two of our members,—Dr. Van Peyma, in educational matters, and Dr. Mann in the work of beautifying this city,—as noteworthy and honorable examples of what physicians may and should do in the rôle of citizens. If individual men can do so much what cannot organised effort emanating from a strong and united medical society accomplish? During the year one of our special meetings was devoted to a discussion of the question of additional milk inspectors and resolutions thereon; another to efforts to defeat the osteopathic bill before the legislature. These are but instances of what an organised society can and should do for the community. Dr. Edward N. Brush, a former resident of this city, in a recent address in Baltimore on the "Physician as a Citizen," remarks:

If the physician is, as a citizen and as a professional man, interested in those things which go for the best training and culture of the coming generation, he is all the more interested in all those matters which affect the life, health and progress of the present, for they are elements which enter into his every-day life and occupation, which must be considered at every bedside. This is eminently the age of preventive medicine. Small-pox, cholera, the plague, malaria, typhus and yellow fever, as familiar conditions in the old days, as are typhoid and pneumonia today, have been stamped out or are under control, and now we are commencing to grapple in an intelligent manner with the white scourge, tuberculosis. If, therefore, there is anything in the lives we lead, in the business or pleasures which we pursue, which leads in our children or in ourselves to mental wreck or moral deterioration, should not the medical profession take cognisance thereof, and, pointing out the danger, suggest the remedy?

We hear now and then of the force of awakened public sentiment. The very phrase implied that public sentiment sometimes sleeps, and while awakening and rubbing its eyes and getting its bearings, your practical politician, who never sleeps, will snatch the prize for which he is scheming,—the control of public affairs, the key of the public treasury. What is needed in this land and in this day is a live, active, wideawake, always vigilant public sentiment, not one which needs awakening or is only aroused into action by some public or official scandal. In this respect no class can set a better example than the members of the profession to which we belong; none, I fear, is more apt to feel that these matters need not engage their attention. We are, I take it, united as to the necessity of clean and well-paved streets, of a pure water supply, of an efficient system of sewerage for our cities and towns, and yet of the hundreds of doctors who realise the importance of these matters, how many personally take the pains to help secure the best men and the most efficient means to accomplish these things?

It is a physician's duty then not only to be a good doctor, but if he will exercise the influence he ought, he should extend his culture and make himself an intelligent force,—a force which exercised through an organised body of medical men is bound to make itself felt. It is my desire to impress the fact that while to know one's business may be the whole duty of the medical man, it is not the whole duty of the cultivated physician; not the whole duty to himself, or to the standing of his profession before the public. "The very term, medical man, in itself illustrates the comparatively low standing of the profession in that mother country of ours, where it is the common term. For our own welfare it behooves us, as students, to prevent any possibility of our descent to the level of a trade, which that term implies. But let not the thought be lost sight of that the duty of the student is, first and foremost, to perfect himself in his knowledge of medicine. No success, no distinction, no fame in arts or letters, or in other fields, can atone for neglect of this. This is the foundation to which his other attainments may be added, but may not supplant. The cultivation of letters, the pursuit of literature, achievements in the sciences allied to medicine,—these not only add directly to the reputation and the influence of the physician who broadens thus his scope, but they add directly to the sum-total of the knowledge of the world. And further still, they stimulate the mental vigor of the intellectual world, and in the gain the physician's own profession reaps its share of benefits as well.

Our academy has many needs, but to have something unattained is to have something to hope for, to spur us on to greater endeavor and activity. We need a permanent meeting place, a home—and for that we have the nucleus of a building fund and fortunately, a growing one. We need a medical library, as Dr. William C. Krauss has so well and ably pointed out in his excellent article of this past winter, or a consolidation of the existing ones. A permanent building of our own would much simplify this question in its solution. In our relations to the community and to questions of public health which arise, from time to time, a standing committee on public health and economics might be desirable and useful. To keep up our interest and stimulate discussion and attendance, first and last of all, I recognise that we must present attractive programs of scientific worth and value, both in general medicine and the specialties, and while I speak of this, I must refer to the benefits of an occasional paper dealing with the honorable history of our profession. An editorial in the *Medical Record* thus refers to the address of Dr. Cordell, of Baltimore, on the value of the study of the history of medicine.

In the first part of his address he criticised somewhat severely the apathy of university and college authorities with regard to the teaching of medical history to medical students. In support of his contention he quotes the saying of Lord Macaulay that "no man, who is correctly informed as to the past, will be disposed to take a morose or desponding view of the present." The speaker especially deprecated the rage for novelty and the haste, characteristic of Americans, and argued that knowledge of the past cannot fail to stimulate the young, and work in every way for good. Dr. Cordell thus summed up some of the advantages of the study of medical history:

(1) It teaches what and how to investigate; (2) it is the best antidote we have against egotism, error, and despondency; (3) it increases knowledge, gratifies natural and laudable curiosity, broadens the view, and strengthens the judgment; (4) it is a rich mine from which may be brought to light many neglected or overlooked discoveries of value; (5) it furnishes the stimulus of high ideals which we poor, weak mortals need to have before us; it teaches our students to venerate what is good, to cherish our best traditions, and strengthens the common bond of the profession; (6) it is the fulfilment of a duty,—that of cherishing the memories, the virtues, the achievements of a class which has benefited the world as no other has, and of which we may feel proud that we are members.

I need but point to the growing number of journal and historical clubs among medical men in this country to show that I am not alone in feeling that a study of the history of the high calling of medicine can but increase our self-respect, our dignity, our standing in the community and our love for one of the noblest of professions.

